# The Connection Between Depression and Ischemic Heart Disease: Analyzing Demographic Characteristics, Risk Factors, Symptoms, and Treatment Approaches to Identify Their Relationship

**DOI:** 10.3390/clinpract14050171

**Published:** 2024-10-17

**Authors:** Laura Ioana Bondar, Brigitte Osser, Gyongyi Osser, Mariana Adelina Mariș, Ligia Elisaveta Piroș, Robert Almășan, Csongor Toth, Caius Calin Miuta, Gabriel Roberto Marconi, Ana-Liana Bouroș-Tataru, Victor Măduța, Dana Tăședan, Mircea Ioachim Popescu

**Affiliations:** 1 Doctoral School of Biomedical Sciences, University of Oradea, 410087 Oradea, Romania; bondar.lauraioana@student.uoradea.ro (L.I.B.); brigitte.osser@uav.ro (B.O.); csongor.toth@uav.ro (C.T.); procardia_oradea@yahoo.com (M.I.P.); 2 Faculty of Physical Education and Sport, “Aurel Vlaicu” University of Arad, 310130 Arad, Romania; caius.miuta@uav.ro (C.C.M.); gabriel.marconi@uav.ro (G.R.M.); 3 Department of Biology and Life Sciences, “Vasile Goldiș” Western University of Arad, 310048 Arad, Romania; tataru.liana@uvvg.ro; 4 Department of General Medicine, “Vasile Goldiș” Western University of Arad, 310048 Arad, Romania; maris.mariana@uvvg.ro (M.A.M.); piros.ligia@uvvg.ro (L.E.P.); almasan.robert@uvvg.ro (R.A.); 5 Arad County Clinical Hospital, 310037 Arad, Romania; maduta.victor@student.uvvg.ro (V.M.); tasedan_dana@yahoo.com (D.T.)

**Keywords:** depression, ischemic heart disease, risk factors, clinical interventions, mental health

## Abstract

**Background:** This study investigates the association between depression and ischemic heart disease (IHD), conditions that often coexist and complicate patient management. Understanding the impact of demographic factors, risk factors, symptoms, and medical approaches in these patients is essential to develop effective clinical strategies. **Objectives:** The aim of this study is to investigate how demographic characteristics, risk factors, symptoms, and treatment methods differ between patients with depression alone and those with both depression and IHD. It seeks to identify how these factors influence patient outcomes, providing insights to improve management and treatment approaches for this complex patient group. **Materials and Methods**: This cross-sectional study included a sample of 332 patients diagnosed with depression, with a specific subgroup consisting of individuals who also had comorbid IHD. Statistical analyses were performed to compare the patients with depression, focusing on those with IHD. Data on demographic characteristics (e.g., gender, environment, social status), risk factors (e.g., hypertension, diabetes), symptom severity, and treatments (e.g., antidepressants, antipsychotics, anxiolytics, hypnotics) were analyzed. The study also evaluated the frequency of cardiac examinations and emergency hospitalizations. **Results:** Significant demographic differences were found between the two groups. Patients with both depression and IHD had higher rates of hypertension and diabetes mellitus and experienced more severe depressive symptoms, including reduced mood, energy, and activity levels. The treatment patterns were similar in terms of antidepressant use, but the IHD group had a higher use of antipsychotics, anxiolytics, and hypnotics. Additionally, these patients required more cardiac examinations and emergency hospitalizations. **Conclusions:** Comorbidity between depression and IHD presents complex clinical challenges, and it is crucial to implement an integrated management approach that addresses both mental and physical health. This study highlights the need for comprehensive therapeutic strategies to improve the quality of life and outcomes for patients with these coexisting conditions.

## 1. Introduction

Depression and IHD are two common and severe chronic diseases that impact global public health. Any number of cases of these diseases would be too high given the substantial burden that depression places on the mental health system and overall quality of life for millions across the world. While IHD continues to represent a substantial public health burden as one of the leading causes of morbidity and mortality, it is also a challenge to cardiovascular health. These concomitant conditions, depression and IHD, are widely considered a difficult clinical picture that increases the severity of the accompanying disease and the difficulty in treating the disease, while at the same time worsening the outcomes [1,2,3]. Notably, recent research demonstrated that depression is associated with an increased risk of developing coronary artery disease. Similarly, depression has been associated with a higher risk of adverse outcomes, including death, in patients with pre-existing cardiovascular disease [4].

Current research is investigating the relationship between the mechanisms of depression and IHD, providing clues suggesting how each may perpetuate or exacerbate the onset, progression, or treatment of the other. For example, depression has been linked to inflammatory responses and lifestyle factors, which increase the risk of IHD, and individuals with depressive symptoms in combination with a cardiovascular disease can experience further psychological stress relating to a chronic disease carrying a poor prognosis. This interaction highlights the need for the correct identification of risk factors, as well as management aimed at a favorable outcome [5,6,7,8]. Several studies highlighted that the prevalence of depression in patients with IHD is significantly higher in women and individuals from lower socioeconomic backgrounds, with these demographic factors further influencing the disease severity and outcomes [9].

Circadian rhythms are fundamental for a variety of physiological and behavioral functions, including sleep, hormone secretion, and mood regulation. Disruptions in these rhythms such as delays, advances, and desynchronization are associated with mental health disorders, particularly major depressive disorder and seasonal affective disorder. Research shows that circadian dysregulation significantly contributes to depression, as evidenced by the efficacy of treatments like novel antidepressants, light therapy, and sleep deprivation interventions [10]. Additionally, circadian rhythms regulated by molecular clocks are vital for cardiovascular health, influencing the endothelial function, thrombus formation, blood pressure, and heart rate. Acute cardiovascular events, including myocardial infarction, stroke, and arrhythmias, also follow specific circadian patterns. Disruptions to these rhythms are associated with cardiovascular diseases and may further exacerbate sleep problems. This bidirectional relationship between circadian biology and cardiovascular health highlights the potential for therapeutic interventions that modify molecular timing, thus enhancing existing treatments and developing new therapies tailored to human physiology [11].

These associations between circadian rhythms, depression, and IHD suggest complex interactions that may exacerbate both disorders. This study demonstrates that there is a mixed picture regarding demographic factors, risk factors, symptoms, and treatment modalities among depressed patients with coexisting IHD. Many studies reported that specific demographics and risk factors affect both the prevalence and severity of the two conditions significantly, while others argue that the clinical profile and comorbidities should be better tailored at an individual level when focusing on one condition [12,13]. Furthermore, individuals with comorbid depression and IHD often experience more severe cardiovascular symptoms, including an increased frequency of angina, higher rates of hospitalizations, and worse prognosis overall [14]. However, much of the existing literature focuses on these disorders in isolation, neglecting the nuances of their interactions when they present concurrently.

This study aimed to investigate the relationship between depression and IHD by using statistical tests to determine the impact of demographic factors, risk factors, symptoms, and treatments. This study evaluates these characteristic factors to provide better patient management, thereby improving outcomes and treatment. Key findings from these results show that demographic characteristics, risk profiles, and symptoms differ by depression and IHD, with significant associations found for each disease state. These consequences emphasize the need for a holistic approach to the treatment of depression with comorbid IHD, as well as individualized care strategies. The presented findings will contribute to a better appreciation of strategies for addressing the combined problem of depression and IHD, thereby guiding improved clinical care and patient outcomes.

The rationale for this study design is the need to better understand the sociodemographic characteristics of patients suffering from both depression and coronary heart disease. Previous research has often focused on either condition in isolation, neglecting the nuances that arise when these diseases coexist. By examining demographic factors such as age, gender, marital status, and socioeconomic status, this study aims to identify specific patient profiles that may be more vulnerable to developing both conditions.

Additionally, the study will use statistical tests to explore the relationship between these demographic characteristics, risk factors, symptoms, and treatment approaches in patients diagnosed with both depression and IHD. The purpose is to evaluate how these factors affect patient management and outcomes. These findings emphasize the necessity of a holistic approach in the treatment of patients with comorbid depression and IHD, advocating for individualized care strategies tailored to the specific needs of the patient. Ultimately, this study aims to improve the understanding and clinical care of patients suffering from the dual challenge of depression and IHD.

## 2. Materials and Methods

### 2.1. Methodological Details

#### 2.1.1. Sample Description

This analysis is based on a cross-sectional study conducted in a population of patients with diagnosed depression either with or without coexisting IHD. The study population consisted of 332 patients admitted to Arad County Emergency Hospital, Psychiatry Department, and diagnosed with depression, who underwent further clinical examination for the presence or absence of IHD. The patients were selected from May 2021 to May 2024 and had the following characteristics:

- Age distribution: The participants’ ages ranged from 40 to 89 years, divided into three distinct age groups: 40–59, 60–79, and 80–89 years.
- Gender distribution: Female, male.
- Marital status: Married, divorced, single, widowed.
- Socioeconomic status: Unemployed, employed, disability pension, age pension, disability pension.

#### 2.1.2. Patient Groups

Therefore, two distinct groups were formed for the analysis:

1. Group 1—149 patients diagnosed with depression without IHD;
2. Group 2—183 patients diagnosed with depression and IHD.

#### 2.1.3. Inclusion and Exclusion Criteria

The inclusion criteria for the study were as follows:

- Adult patients.
- Patients admitted to the Psychiatry Department of the Arad County Emergency Clinical Hospital.
- Confirmed diagnosis of depression fulfilling the International Classification of Diseases—10 (ICD-10) criteria.
- Availability of complete medical records.
- A willingness of the patient to participate in the study.

The exclusion criteria for the study were as follows:

- Incomplete medical records.
- Other significant comorbidities unrelated to the scope of the study that may produce misleading results (e.g., severe malignancies, neurological disorders).
- Patients’ unwillingness to participate in the study.

#### 2.1.4. Data Collection

Data were collected using comprehensive medical records including the following parameters:

- Demographic data: Age, gender, environment, marital status, and socioeconomic status.
- Medical history:
  ○ A history of previous depressive episodes.
  ○ A history of IHD.
  ○ A family history of depression or cardiovascular diseases.
- Comorbidities: The presence of other mental health disorders (e.g., anxiety) and physical health conditions (e.g., hypertension, diabetes, tachycardia).
- Risk factors:
  ○ Lifestyle factors (e.g., smoking, alcohol, coffee consumption).
  ○ Biological risk factors (e.g., obesity, metabolic syndrome, hypercholesterolemia, hypertriglyceridemia, dyslipidemia, inflammation, genetic factors).
- Depressive symptoms: Evaluated using standardized assessment tools such as the Beck Depression Inventory or Hamilton Depression Rating Scale, along with direct patient investigation.
- Treatment modalities: The type of medication prescribed (e.g., antidepressants, antipsychotics, mood stabilizers, anxiolytics, hypnotics) and other interventions.

#### 2.1.5. Testing Methods

An additional method used for testing patients involved the administration of a comprehensive clinical assessment at the initial evaluation stage. This assessment was conducted to ensure an accurate diagnosis and to gather baseline data on depressive symptoms and physical health status. Patients underwent both psychological evaluations and physical examinations, including electrocardiograms and routine laboratory tests, to assess their cardiovascular health.

#### 2.1.6. Statistical Analysis

The main variables of interest in this analysis were the presence or absence of IHD, the various risk factors, and depressive symptoms, which were all categorized and coded for the statistical analysis.

The association of the variables regarding depression alone and depression combined with IHD was performed using binomial and multinomial tests. These tests were selected due to their effectiveness in comparing two groups regarding the proportion to the outcomes of interest. For the binomial test, the significance for all those variables was tested at the 95% confidence level, taking *p*-values below 0.05 to be significant. The reason for using 0.5 is that it represents the most conservative estimate in the absence of strong prior information or assumptions. By assuming equal probability for both outcomes (i.e., 50/50), the binomial test maximizes the uncertainty, thereby providing a rigorous and neutral basis for hypothesis testing. For multiple variables, the multinomial test was utilized, as it is specifically designed for analyzing probabilities across multiple categories.

Data analysis was performed using JASP software, and the results were presented as proportions with the corresponding *p*-values. For variables with multiple levels, comparisons were made between the individual levels within each group.

#### 2.1.7. Ethical Approval

This study received approval from the Ethics Committee of Arad County Clinical Hospital under approval code 38/6 April 2021. All patient data were anonymized to ensure confidentiality, and the study followed the ethical standards of the Declaration of Helsinki. The raw data used and analyzed during the current study are available from the authors on request.

### 2.2. Hypotheses of the Study

This study is based on the following hypotheses that serve as the foundation for investigating the association between depression and IHD:

1. Demographic differences: There are significant differences in demographic characteristics (age, gender, marital status, socioeconomic status) between patients with depression alone and those with comorbid depression and IHD.
2. Risk factor association: Patients diagnosed with both depression and IHD have a higher prevalence of risk factors (hypertension, diabetes, lifestyle factors) compared to patients with depression only.
3. Symptom severity: Individuals with comorbid depression and IHD experience more severe depressive symptoms (decreased mood, energy, and activity levels) than those with depression alone.
4. Treatment: The treatment approach for patients with comorbid depression and IHD differs significantly from those with depression alone, with a higher utilization of antipsychotics, anxiolytics, and other medications in the former group.
5. Holistic management needs: The coexistence of depression and IHD requires a comprehensive approach to management and treatment, which improves patient outcomes when individualized care strategies are implemented.

These hypotheses will be tested through statistical analysis, and the results will provide insight into the relationships between demographic factors, risk factors, symptoms, and treatment approaches for patients with both disorders. The findings aim to contribute to the understanding of effective management strategies for patients with comorbid depression and IHD.

## 3. Results

The results of the current study have several important implications for the associations between depression and IHD, showing different risk factors, symptoms, and treatment modalities. This study aims to enhance our comprehension of this relationship by conducting statistical tests that could deepen our insight into how these factors contribute to the overall health outcomes in patients suffering from both depression and IHD.

The diagnosis of depression was established following the ICD-10 criteria, including a comprehensive process that involves both the patient’s self-reported history and information from close relatives. Additional data were collected from observation files to pro-vide a more complete clinical picture. A detailed analysis of the patient’s personal life history, disease progression, and family background was also performed. To improve the diagnostic accuracy, standardized assessment tools such as the Beck Depression Inventory and the Hamilton Depression Rating Scale were used. These scales are widely recognized in clinical practice to assess the severity of depressive symptoms and confirm the presence of the disorder. The use of these diagnostic methods ensures a comprehensive approach that combines subjective and objective data, providing a clear and reliable diagnosis of depression.

### 3.1. Statistical Analysis of Gender and Environmental Factors in Patients with Depression Alone Compared to Those with Comorbid IHD

Table 1 compares gender and environmental factors between the two groups: patients diagnosed with depression only (*n* = 149) and those with both depression and IHD (*n* = 183). The variables investigated include age, gender, environment, and marital and social status, with significance indicated by *p*-values. The main findings are as follows:

- Gender
  ○ Depression only: Females constitute 61.7% of this group, with a statistically significant difference compared to males (38.3%, *p* = 0.005).
  ○ Depression + IHD: The gender distribution is more balanced, with 54.1% males and 45.9% females, indicating no significant difference between the genders in the comorbidity group (*p* = 0.301). This suggests a change in the gender distribution in the presence of IHD.
- Environment
  ○ Depression only: The majority of patients are from urban areas (61.7%, *p* = 0.005) compared to the 38.3% from rural areas.
  ○ Depression + IHD: No significant difference was found in the distribution between urban (55.7%) and rural (44.3%) environments (*p* = 0.139), indicating that the presence of IHD may change the environmental distribution occurring in depression alone.

### 3.2. Statistical Results for Evaluating Age, Social Status, Marital Status, Diagnosis, and Severity in Patients with Depression Alone Versus Depression with Comorbid IHD

Table 2 compares various factors such as age, social status, marital status, diagnosis, and severity between patients diagnosed with depression only (*n* = 149) and those with both depression and IHD (*n* = 183) to determine whether there are significant differences between these factors in both groups. The specific breakdown of the key statistics is as follows:

- Age
  ○ Depression only: The multinomial test revealed a significant result: χ^2^ = 25.973, df = 1, and *p* < 0.001. This indicates a strong association between age and depression, meaning that age has a statistically significant effect on the development of depression.
  ○ Depression + IHD: The results were even more significant: χ^2^ = 81.738, df = 2, and *p* < 0.001. This shows that age is also highly significant in this group and varies more, as the higher degrees of freedom indicate.
- Social status
  ○ Depression only: χ^2^ = 32.811, df = 4, and *p* < 0.001. This suggests that social status has a significant relationship with depression, with varying levels of social status contributing to the condition.
  ○ Depression + IHD: The relationship was even stronger: χ^2^ = 54.732, df = 4, and *p* < 0.001. This indicates that social status is a key factor affecting patients with both conditions.
- Marital status
  ○ Depression only: Marital status had an extremely strong association with depression: χ^2^ = 298.919, df = 3, and *p* < 0.001. This suggests that one’s marital status has a significant impact on the development of depression.
  ○ Depression + IHD: A similarly strong association was found in patients with both depression and IHD: χ^2^ = 144.978, df = 3, and *p* < 0.001. Marital status appears to be an important variable in both groups.
- Psychiatric diagnosis
  ○ Depression only: The multinomial test for the diagnosis variable in the depression-only group showed that χ^2^ = 97.459, df = 3, and *p* < 0.001. This suggests that different diagnostic factors significantly influence depression.
  ○ Depression + IHD: The relationship remains significant: χ^2^ = 108.388, df = 3, and *p* < 0.001. This suggests that diagnosis also plays an important role in the dual condition of depression and IHD.
- Grade:
  ○ Depression only: The multinomial result was that χ^2^ = 14.284, df = 2, and *p* < 0.001, indicating a significant association between the severity of depression and the condition.
  ○ Depression + IHD: The significance remained: χ^2^ = 11.836, df = 2, and *p* = 0.003. This is a slightly higher *p*-value, but still indicating a strong relationship between the degree and the combined conditions.

#### 3.2.1. Age-Related Differences in Depression With and Without Comorbid IHD

**Figure 1:**
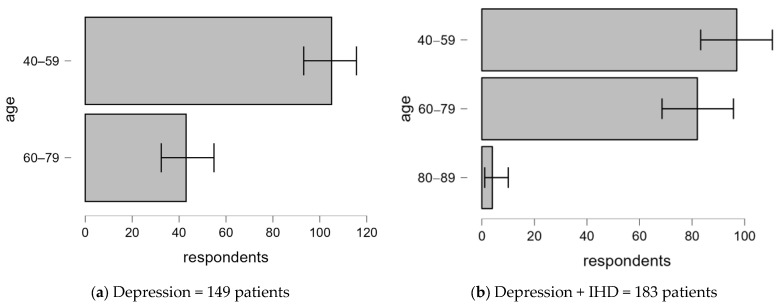
Age distribution of patients with depression alone compared to those with comorbid IHD.

Figure 1 presents the age profile of patients diagnosed with depression alone compared to those with comorbid IHD.

Depression only: The majority of patients (70.5%) fall into the 40–59 age group, while patients aged 60–79 years (29.5%) are significantly underrepresented, and none are included in the 80–89 age group.
Depression + IHD: The age distribution changes significantly in this group, with a higher proportion of patients aged 60–79 (44.8%). There is a small representation of patients aged 80–89 (2.2%). This suggests that older age is more associated with comorbid depression and IHD.

#### 3.2.2. Social Status-Related Differences in Depression With and Without Comorbid IHD

**Figure 2:**
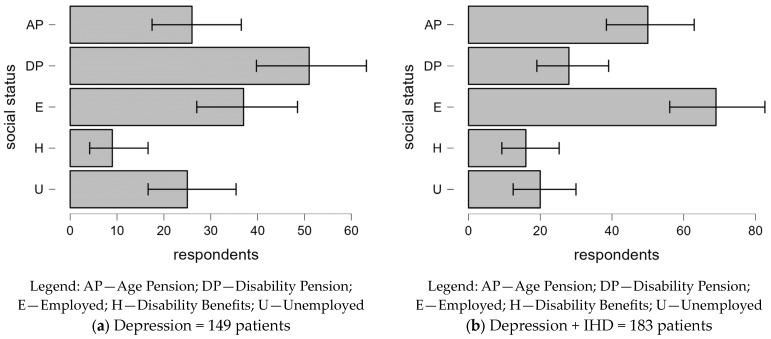
Social status distribution of patients with depression alone compared to those with comorbid IHD.

Figure 2 illustrates the social status of patients diagnosed with depression alone compared to those with comorbid IHD:

- Depression only: Most patients receive a disability pension (34.2%) or are employed (25.5%). In contrast, the unemployed (16.8%) and those on disability benefits (6%) are notably underrepresented (*p* < 0.001 across all categories).
- Depression + IHD: There is a marked increase in the proportion of employed individuals (37.7%) and those on age pension (27.3%) compared to those with disability pension (15.3%) or who are unemployed (10.9%). This indicates that the social status distributions vary significantly with the presence of IHD, possibly reflecting changes in employment status due to the added burden of chronic illness.

#### 3.2.3. Marital Status-Related Differences in Depression With and Without Comorbid IHD

**Figure 3:**
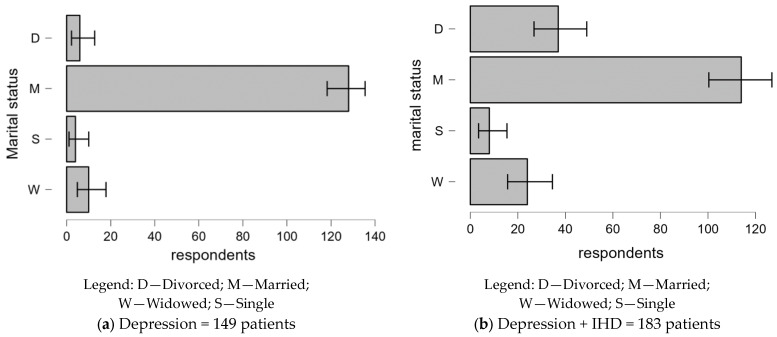
Marital status distribution of patients with depression alone compared to those with comorbid IHD.

Figure 3 compares the marital status of patients diagnosed with depression alone in comparison to those with comorbid IHD:

- Depression only: Married patients dominate this group (86.6%), while widowed (6.7%), divorced (4%), and single (2.7%) patients are significantly underrepresented.
- Depression + IHD: There is a significant increase in divorced (20.2%) and widowed (13.1%) patients, with a corresponding decrease in married individuals (62.3%). This suggests that marital status, especially being divorced or widowed, is more common in patients with both conditions.

#### 3.2.4. Differences in Diagnostic Categories of Depression With and Without Comorbid IHD

**Figure 4:**
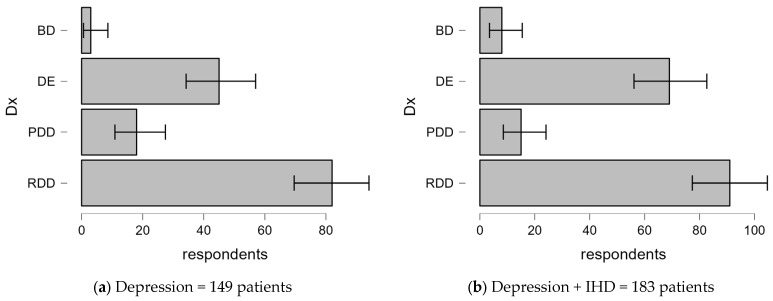
Distribution of diagnostic categories in patients with depression alone compared to those with comorbid IHD.

Figure 4 shows the diagnostic categories of patients diagnosed with depression alone compared to those with comorbid IHD.

- Bipolar Disorder (BD):
  ○ Depression only: In total, 2% of patients were diagnosed with BD.
  ○ Depression + IHD: The prevalence increases to 4.4%. This suggests a slightly higher occurrence of BD among patients with both conditions.
- Depressive Episode (DE):
  ○ Depression only: A total of 30.9% of patients had DE.
  ○ Depression + IHD: A higher proportion of 37.7% was observed. This indicates that depressive episodes are more prevalent in those with comorbid IHD.
- Persistent Depressive Disorder (PDD):
  ○ Depression only: In total, 12.1% of patients were diagnosed with PDD.
  ○ Depression + IHD: A slight decrease of 8.2% was found, suggesting a lower association between PDD with IHD.
- Recurrent Depressive Disorder (RDD):
  ○ Depression only: This was the most common diagnosis, affecting 55% of patients.
  ○ Depression + IHD: The prevalence of RDD remained high at 49.7%, showing no significant difference compared to the depression-only group.

#### 3.2.5. Differences in Depression Severity With and Without Comorbid IHD

**Figure 5:**
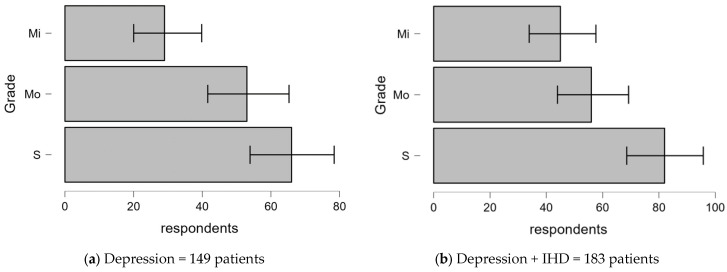
Distribution of depression grades in patients with depression alone compared to those with comorbid IHD.

Figure 5 illustrates the severity of depression in patients diagnosed with depression alone compared to those with comorbid IHD.

- Mild (Mi):
  ○ Depression only: In total, 20.1% of patients had mild depression.
  ○ Depression + IHD: The proportion increased, indicating a trend towards mild severity in comorbid patients.
- Moderate (Mo):
  ○ Depression only: A total of 35.6% of patients experienced moderate depression.
  ○ Depression + IHD: This decreased slightly to 30.6%. This may reflect the impact of IHD on altering the distribution of depression severity.
- Severe (S):
  ○ Depression only: Severe depression was present in 44.3% of patients, with no significant result.
  ○ Depression + IHD: The prevalence was similar at 44.8%, indicating that depression severity remained consistently high across both groups.

### 3.3. Statistical Results for Evaluating the Association of Risk Factors in Patients with Depression Alone Versus Depression with Comorbid IHD

The binomial test statistics, reported in Table 3, show the association of comorbidities and various health factors in patients diagnosed with depression only (*n* = 149) compared to patients with both depression and IHD (*n* = 183). The breakdown of the key statistics is presented below.

- Comorbidities:
  ○ Depression only: In total, 82.6% of patients had comorbid conditions (*p* < 0.001).
  ○ Depression + IHD: A total of 83.6% also reported comorbidities (*p* < 0.001). The high prevalence in both groups suggests that many patients with depression face additional health challenges, highlighting the importance of a comprehensive treatment approach.
- Hypertension:
  ○ Depression only: A total of 61.1% of patients had hypertension (*p* = 0.009).
  ○ Depression + IHD: A significantly higher 93.4% had hypertension (*p* < 0.001). This suggests that hypertension is a common risk factor among patients with IHD, indicating a potential interaction between these conditions.
- Diabetes Mellitus:
  ○ Depression only: In total, 32.9% of the patients had diabetes.
  ○ Depression + IHD: A total of 54.6% of patients had diabetes, reflecting a higher prevalence of diabetes among IHD patients. This highlights the need for integrated management strategies that address both diabetes and mental health.
- Hypercholesterolemia:
  ○ Depression only: Among patients diagnosed with depression alone, 53% had hypercholesterolemia (*p* = 0.512).
  ○ Depression + IHD: Conversely, a significantly higher proportion of patients (73.2%) exhibited hypercholesterolemia (*p* < 0.001). This significant increase in hypercholesterolemia among patients with both conditions suggests that individuals suffering from comorbid IHD are at greater risk of elevated cholesterol levels. This increase could exacerbate cardiovascular complications and underscores the necessity for vigilant monitoring and management of cholesterol levels in this population.
- Hypertriglyceridemia:
  ○ Depression only: The prevalence of hypertriglyceridemia among patients with depression only was 48.3% (*p* = 0.743).
  ○ Depression + IHD: The prevalence of patients with both depression and IHD increased to 68.3.% (*p* < 0.001). The increase in hypertriglyceridemia in the comorbid group highlights its potential role as a significant risk factor for worsening cardiovascular health. This elevated level of triglycerides among patients with both conditions indicates a critical area for clinical intervention, as addressing HTG could be vital for improving cardiovascular health and overall management in these patients.
- Dyslipidemia:
  ○ Depression only: A total of 37.6% of patients had dyslipidemia.
  ○ Depression + IHD: The prevalence increased to 68.3% (*p* < 0.001). This significant difference suggests that dyslipidemia is more likely in patients with IHD, emphasizing the importance of lipid treatment in these populations.
- Obesity:
  ○ Depression only: In total, 24.2% of the patients were classified as obese.
  ○ Depression + IHD: The proportion of obese patients increased to 63.9% (*p* < 0.001). This highlights that obesity is a significant risk factor for IHD, suggesting that weight management may play an important role in improving outcomes for these patients.
- Metabolic syndrome:
  ○ Depression only: In total, 21.5% of patients were diagnosed with metabolic syndrome.
  ○ Depression + IHD: The prevalence increased to 67.8%, indicating a clear association between metabolic syndrome and IHD (*p* < 0.001). This finding suggests the need for health interventions focused on controlling metabolic risk factors.
- Smoking:
  ○ Depression only: A total of 69.1% of patients were smokers (*p* < 0.001).
  ○ Depression + IHD: A slightly smaller proportion, 67.2%, were smokers (*p* < 0.001). The rates were relatively similar, indicating that smoking cessation efforts may be equally important in both patient groups.
- Alcohol consumption:
  ○ Depression only: A total of 22.1% reported alcohol consumption.
  ○ Depression + IHD: In total, 33.3% reported alcohol use. This difference may indicate a higher tendency of alcohol use among individuals with comorbid IHD, emphasizing the importance of addressing this in treatment plans.
- Coffee consumption:
  ○ Depression only: Among patients diagnosed with depression alone, 91.9% reported drinking coffee (*p* < 0.001).
  ○ Depression + IHD: In contrast, 91.3% of patients with both depression and IHD also reported coffee consumption (*p* < 0.001). The similar rates of coffee consumption in both groups suggests that coffee drinking is a common habit among individuals experiencing depression, regardless of whether they also have IHD. This high level of consumption may indicate that patients use coffee as a coping mechanism or source of comfort, despite its potential implications for cardiovascular health.
- Drug Consumption:
  ○ None of the participants in the sample were taking drugs (100% with *p*-value < 0.001).
- Presence of inflammation:
  ○ Depression only: A total of 40.9% of patients showed signs of inflammation (*p* = 0.033).
  ○ Depression + IHD: This proportion increased significantly to 72.7% (*p* < 0.001), suggesting that inflammation may play an important role in the pathophysiology of both conditions, further complicating the treatment strategy.
- Tachycardia:
  ○ Depression only: Among the patients diagnosed with depression alone, 41.6% experienced tachycardia.
  ○ Depression + IHD: A total of 57.4% of patients suffering from both depression and IHD reported suffering from tachycardia. The prevalence of tachycardia was significantly higher in patients with comorbid depression and IHD compared to those with depression alone. This suggests that the presence of IHD may exacerbate the incidence of tachycardia among individuals who are already suffering from depression.
- Genetic factors:
  ○ Depression only: In total, 79.2% had genetic factors (*p* < 0.001).
  ○ Depression + IHD: The prevalence was lower at 63.4% (*p* < 0.001). This may suggest a complex interplay of genetic and environmental factors influencing the presence of IHD in addition to depression.
- Absent Risk Factors:
  ○ There were no participants without risk factors (100% with *p*-value < 0.001).

### 3.4. Statistical Results for Evaluating the Impact of Symptoms in Patients with Depression Alone Versus Depression with Comorbid IHD

Table 4 focuses on the presence of various psychiatric symptoms and the comparison between patients with depression only and those with depression and IHD. Below is a breakdown of the main findings.

- Low mood: The majority of both groups exhibited low mood, with 91.9% of patients with depression alone and 97.8% of patients with depression and IHD reporting this symptom (*p* < 0.001). This indicates that low mood is a common feature in both populations but is slightly more common in patients with comorbid IHD.
- Low energy: Low energy was reported by 78.5% of patients with depression alone and 88% of patients with depression and IHD (*p* < 0.001). The higher percentage in the comorbid group suggests that IHD may exacerbate feelings of fatigue.
- Low activity: A total of 85.2% of the patients with depression reported low activity levels, compared to 98.9% in the depression and IHD group (*p* < 0.001). This significant difference indicates that comorbid IHD is associated with significantly reduced activity levels in affected individuals.
- Incapacity: In total, 78.5% of patients with depression reported feelings of incapacity, while this was the case for 71% of those with depression and IHD (*p* < 0.001). The data suggest that while both groups experience incapacity, it is slightly more pronounced in patients with depression alone.
- Uselessness: A notable 59.7% of patients with depression felt a sense of uselessness, compared to 77% in the comorbid group. This suggests that individuals with both depression and IHD may struggle more significantly with self-worth issues.
- Guilt: Feelings of guilt were reported by 49.7% of patients with depression alone and 49.2% of those with comorbid IHD, indicating that guilt is a prevalent issue in both populations without a significant difference.
- Worthlessness: Feelings of worthlessness were reported by 63.1% of patients with depression and 73.2% of patients with IHD, suggesting a more profound impact of comorbid IHD on feelings of self-worth.
- Anhedonia: In total, 53% of patients with depression (*p* = 0.512) experienced anhedonia, while this symptom was reported by 74.3% of patients with depression and IHD (*p* < 0.001). This suggests that the presence of IHD is significantly correlated with a loss of interest and pleasure in previously enjoyed activities.
- Isolation: A total of 57.7% of patients with depression (*p* = 0.071) reported feelings of isolation, in contrast to 95% of the depression and IHD group (*p* < 0.001). This underscores an increased sense of social disconnection among patients with both disorders.
- Low self-esteem: Low self-esteem was reported by 63.1% of patients with depression alone and 73.2% of those with IHD, indicating a higher prevalence in the comorbid group.
- Rumination: Rumination was observed in 79.2% of patients with depression compared to 86.3% of those with depression and IHD, suggesting that a tendency to engage in negative thinking may be increased in the comorbid population (*p* < 0.001).
- Lability: Emotional instability was markedly more pronounced in patients with depression who also had IHD. Specifically, 96.2% of individuals with both conditions reported emotional instability, compared to 92.6% of those with depression only (*p* < 0.001).
- Cognitive impairment: Cognitive impairment was reported in 58.3% of patients with depression (*p* = 0.059) and 76.5% of the comorbid group (*p* < 0.001), indicating a significant increase in cognitive difficulties associated with IHD.
- Insomnia: Insomnia was prevalent in 66.4% of those with depression alone and 72.1% of those with depression and IHD, pointing to the possibility that IHD may contribute to sleep disturbances (*p* < 0.001).
- Low appetite: A stark contrast was observed in appetite changes, with 25.5% of patients with depression alone experiencing low appetite versus 93.4% in the depression and IHD group, indicating a profound impact on eating behaviors in those with comorbid conditions.
- Somatic symptoms: Somatic symptoms were prevalent in both patient groups, with 64.4% of those with depression alone and 62.8% of those with comorbid IHD reporting such symptoms, suggesting that the presence of IHD does not significantly increase somatic complaints in patients with depression.
- Weight loss: Similar to appetite, weight loss was reported by 25.5% of patients with depression and a striking 93.4% among those with depression and IHD.
- Low libido: A total of 55% of patients with depression reported low libido (*p* = 0.251), compared to 72.1% in the comorbid group (*p* < 0.001), suggesting that IHD may further diminish sexual desire.
- Suicidal thoughts: Suicidal thoughts were reported in 16.1% of patients with depression alone and 46.4% of those with IHD, highlighting a concerning increase in suicidal ideation associated with comorbidity.
- Delusions: The presence of delusions was reported in 10.7% of patients with depression alone, compared to 24% in those with depression and IHD, indicating a higher risk of psychotic features in the comorbid group.
- Hallucinations: In total, 9.4% of patients with depression experienced hallucinations, in contrast to 6% of those with both depression and IHD, suggesting a significantly greater incidence of perceptual disturbances in the depression-only group.
- Anxiety: A total of 58.4% of patients with depression alone experienced anxiety (*p* = 0.049), as did 89.6% (*p* < 0.001) of those with IHD, suggesting that anxiety is a prominent feature in patients in the comorbid group.

### 3.5. Statistical Results for Evaluating the Impact of Treatment, Cardiological Examination, and Emergency Admission Hospitalization in Patients with Depression Alone Versus Depression with Comorbid IHD

Table 5 analyzes the data of variables reported regarding treatment and medical interventions between patients with depression alone and those with depression and IHD. The following is a breakdown of the main statistics:

- Antidepressants: All patients in both the depression-only group and the depression-with-IHD group received antidepressant treatment, underscoring the fundamental role of these medications in managing depressive symptoms across both populations.
- Antipsychotics: Antipsychotics were used more frequently in patients with IHD, with 35.5% receiving this medication compared to only 14.1% of patients with depression alone.
- Mood stabilizers: A total of 61.1% of the patients with depression alone (*p* = 0.008) and 77.6% (*p* < 0.001) of those with depression and IHD used mood stabilizers. This indicates a greater need for mood regulation in the presence of cardiovascular conditions.
- Anxiolytics: In total, 58.4% of patients with depression alone (*p* = 0.049) were prescribed anxiolytics, compared to a significantly higher 89.6% (*p* < 0.001) in those with depression and IHD. This reflects increased insecurity in the depression-and-IHD population.
- Hypnotics: A total of 66.4% of patients with depression alone and 72.1% of those with depression and IHD used hypnotics, underscoring the commonality of sleep disturbances in both groups (*p* < 0.001).
- Other treatments: A total of 85.9% of patients with depression alone and 87.4% with depression and IHD received other treatments, suggesting a comprehensive approach to managing their conditions (*p* < 0.001).
- Cardiological examination: Only 49.7% (*p* = 1.000) of patients with depression alone underwent cardiological examinations, compared to 77.6% of those with depression and IHD (*p* < 0.001), highlighting the importance of cardiovascular monitoring in this higher-risk group.
- Emergency admissions: A notable 54.4% of patients with depression and IHD required emergency admissions, mirroring the percentage in those with depression alone, which highlights the acute health challenges faced by both groups.

## 4. Discussion

### 4.1. Overview of Findings

The purpose of this study is to conduct a comprehensive analysis of how various factors influence the symptoms, management, and outcomes of patients diagnosed with depression alone and in association with IHD, in alignment with findings from similar studies [15,16]. The findings underline important differences and similarities in demographic characteristics, risk factors, symptomatology, and treatment approaches between these two patient groups. Recent studies emphasize that these insights are essential for developing targeted interventions to address the complex needs of patients dealing with both psychiatric and cardiovascular conditions [17,18].

Our findings align with recent studies suggesting that demographic variables such as gender, socioeconomic status, and living environment are important associations in shaping the clinical and therapeutic profile of these patients [19]. Moreover, significant differences between the two groups in terms of the severity of symptoms and resorting to medical interventions suggest the need for an integrated approach to treatment.

In this discussion section, we will elaborate on the implications of these findings, exploring how IHD features alter the clinical presentation of depression, how comorbidities influence strategies in treatment, and the wider implications for patient care and outcomes. By examining these factors, our aim, consistent with other studies, was to provide a clearer understanding of the challenges associated with treating patients with depression in relation to IHD, thereby promoting further research and advancements in clinical practice [20,21].

### 4.2. Demographic and Diagnostic Characteristics

The demographic and diagnostic characteristics analyzed in patients with depression in comparison with patients with depression and IHD reveal several notable associations. The age distribution is significantly different between the depression-alone and depression-with-IHD groups. Depression is significantly more common in the 40–59 age group compared to the 60–79 age group, suggesting that depression is more common in the middle-aged population. This finding aligns with previous research indicating that the onset of depression is often linked to midlife stressors, including career pressures and family responsibilities [22]. In contrast, the proportion of patients diagnosed with both depression and IHD is more balanced in the 40–59 and 60–79 age groups, with no significant differences in either age group. The absence of patients in the 80–89 age group within the depression-only cohort suggests that the combination of depression and IHD is more likely to affect the elderly population, which is consistent with the literature suggesting that comorbidities are more common in older adults [23].

For comparison, the obtained data on depression provide evidence of a higher number of females, urban residents, and subjects with disabilities or unemployment. Recent studies have often confirmed that women are at a higher risk of depression [24,25]. This suggests that gender, socioeconomic status, and living conditions may be associated with the prevalence and diagnosis of depression. The literature highlights how socioeconomic status influences both depression and comorbidities [26,27,28].

In contrast, patients with both depression and IHD have a more balanced gender distribution and a higher proportion of people living in rural areas. Such a variation may be due to the widespread impact of IHD on the specific health needs of the different demo-graphic groups and comorbid patients. The distribution of diagnostic categories also differs. Patients with comorbid depression and IHD have a higher incidence of recurrent depressive disorder and a slightly different distribution of depression levels compared to patients with depression alone. This would imply that the complexity of treating both disorders may affect the presentation and classification of depressive episodes. Various studies have shown how marital status affects depression, supporting the relevance of this factor in this study [29,30].

### 4.3. Risk Factors and Comorbidities

A critical aspect of this study was the comparison of risk factors and comorbidities between the two patient groups. Although hypertension was commonly observed in both groups, the prevalence and type of comorbidities differed significantly. Patients with depression and IHD were more likely to suffer from comorbidities such as dyslipidemia, obesity, and metabolic syndrome. These findings are consistent with recent research, underscoring the importance of comprehensive treatment strategies that address both psychiatric and cardiovascular health [31].

Interestingly, there were also differences in the prevalence of diabetes and hypercholesterolemia between the two groups, suggesting a possible interplay between metabolic factors and dual diagnosis, which could be indicative of the underlying pathophysiological mechanisms or different effects of treatment and lifestyle on these patients. The current findings reiterate that comorbid conditions such as hypertension and diabetes worsen depression [32]. Research has highlighted the importance of addressing multiple comorbidities for effective treatment, which is consistent with the findings of this study [33].

### 4.4. Symptomatology

When depressive symptoms were analyzed, patients with both depression and IHD exhibited higher rates of severe depressive symptoms, including low mood, low energy, and anhedonia, compared to patients with depression alone. This trend emphasizes that IHD exacerbates the severity of depressive symptoms, indicating a need for simultaneous treatment of both disorders [34].

While symptoms such as guilt, worthlessness, and cognitive impairment seem to be of similar frequencies in both groups, symptoms such as hallucinations and suicidal ideation are increased in the comorbid group. This may indicate that certain symptoms are more severe or are treated differently in serious physical illnesses like IHD. Studies support the higher prevalence of symptoms such as insomnia in exacerbations with comorbid depression and IHD. Several studies have found that the severity of symptoms differs between patients with depression alone and those with comorbid IHD, which mirrors the results of this study [35,36].

### 4.5. Treatment Approaches

The analysis of medications and medical interventions shows that both patient groups receive antidepressants in a fairly similar way, reflecting the standard treatment of depression. However, the use of other medications and interventions differ. Patients with both depression and IHD are more likely to receive antipsychotics, mood stabilizers, and anxiolytics, indicating greater needs for complex psychiatric treatment in this group. This may be because of the higher severity and complexity in the treatment of comorbid conditions, which call for higher prescription rates of these medications. Recent studies in antidepressant use trends and variability in the use of additional medications report findings that are consistent with this article, suggesting a consistent approach to the use of antidepressants and variability in the use of other treatments [37,38].

In addition, patients with comorbid depression and IHD have more frequent cardiological examinations and emergency interventions. The higher intervention rate points to the fact that careful follow-up and timely treatment are obligatory in these high-risk cases involving both depression and cardiovascular diseases. In this regard, the literature often refers to the accompaniment of cardiological testing regarding the management of depression combined with IHD [39,40].

### 4.6. Implications for Patient Care

The findings of this study underscore the necessity for an integrated approach to treating patients with both depression and IHD. Given the significant differences in demographic characteristics, symptom severity, and treatment needs, healthcare providers must develop targeted interventions that address the unique challenges faced by these patients [41].

### 4.7. Research Limitations

Despite the valuable insights gained from this study, several limitations must be acknowledged. One significant limitation is the potential risk of selection bias. Although efforts were made to mitigate this risk by using comprehensive medical records and conducting direct patient examinations, the possibility of bias cannot be entirely eliminated. Additionally, the cross-sectional design of this study fundamentally restricts the ability to draw causal relationships between variables.

Future research should consider implementing longitudinal studies to evaluate the long-term impacts of comorbid conditions on patient outcomes. A broader geographic and demographic representation would also improve the generalizability of the results. Therefore, these findings should be validated in future studies to confirm the findings obtained in this research.

### 4.8. Clinical Implications and Recommendations for Future Research

The findings of this study highlight the need for an integrated approach to treating patients with both depression and IHD. Given the significant differences in demographic characteristics, symptom severity, and treatment needs, healthcare providers must develop targeted interventions that address the unique challenges faced by these patients.

To further enhance the discussion, the following clinical implications and recommendations are proposed:

- Longitudinal studies: Future research should focus on longitudinal studies to evaluate the long-term effects of comorbid conditions on patient outcomes and treatment efficacy.
- Diverse populations: Expanding research to include diverse populations, particularly different ethnic and socioeconomic groups, will improve the generalizability of the results.
- Integrated treatment models: Investigating integrated treatment models that simultaneously address both psychiatric and cardiovascular needs may improve patient outcomes.
- Psychoeducation and support: Developing psychoeducation and support programs for patients and caregivers can help manage the complexities of living with both depression and IHD.
- Exploring mechanisms: Future studies should investigate the biological and psychological mechanisms linking depression and IHD to provide targeted interventions.

## 5. Conclusions

This study examines the impact of demographic factors, risk factors, symptoms, and medical interventions on patients diagnosed with depression with or without comorbid IHD. The findings reveal that the presence of IHD not only exacerbates the clinical presentation of depression but also necessitates a more individualized and comprehensive treatment approach.

Key results indicate that patients with comorbid depression and IHD experience higher severity of depressive symptoms, a greater prevalence of specific comorbidities, and an increased need of complex psychiatric interventions compared to those with depression alone. These findings highlight the critical importance of integrating psychiatric and cardiovascular care, thereby addressing the intricate health needs of this vulnerable patient population.

Furthermore, this study highlights the necessity of recognizing demographic variables such as age, gender, and socioeconomic status as influential factors in the clinical and therapeutic profiles of patients with comorbid conditions. The demonstrated differences in treatment approaches (especially the increased prescription rates of antipsychotics, mood stabilizers, and anxiolytics in the comorbid group) reinforce the need for tailored management strategies.

Moving forward, further research is essential to elucidate the interactions between depression and IHD, ultimately optimizing treatment protocols and improving patient outcomes. This study provides valuable insights that can inform clinical practice and guide future investigations aimed at enhancing the quality of care for patients facing the dual challenges of depression and IHD.

## Figures and Tables

**Table 1:** Binomial test application for evaluating gender and environmental factors in patients with depression alone versus depression with comorbid IHD.

		Depression = 149 Patients		Depression + IHD = 183 Patients	
Variable	Level	Counts	*p*	Counts	*p*
Gender	F	92 (61.7%)	0.005	84 (45.9%)	0.301
	M	57 (38.3%)	0.005	99 (54.1%)	0.301
Environment	R	57 (38.3%)	0.005	81 (44.3%)	0.139
	U	92 (61.7%)	0.005	102 (55.7%)	0.139

F—female, M—male, R—rural, U—urban.

**Table 2:** Multinomial test application for evaluating age, social status, marital status, diagnosis, and severity on patients with depression alone versus depression with comorbid IHD.

	Depression = 149 Patients			Depression + IHD = 183 Patients		
Variable	χ^2^	df	*p*	χ^2^	df	*p*
Age	25.973	1	<0.001	81.738	2	<0.001
Social status	32.811	4	<0.001	54.732	4	<0.001
Marital status	298.919	3	<0.001	144.978	3	<0.001
Psychiatric diagnosis	97.459	3	<0.001	108.388	3	<0.001
Grade	14.284	2	<0.001	11.836	2	0.003

df—degree of freedom.

**Table 3:** Binomial test application for evaluating the associations of the risk factors with patients with depression alone versus depression with comorbid IHD.

		Depression = 149 Patients		Depression + IHD = 183 Patients	
Variable	Level	Counts	*p*	Counts	*p*
Comorbidities	No	26 (17.4%)	<0.001	30 (16.4%)	<0.001
	Yes	123 (82.6%)	<0.001	153 (83.6%)	<0.001
HTN	No	58 (38.9%)	0.009	12 (6.6%)	<0.001
	Yes	91 (61.1%)	0.009	171 (93.4%)	<0.001
DM	No	100 (67.1%)	<0.001	83 (45.4%)	0.237
	Yes	49 (32.9%)	<0.001	100 (54.6%)	0.237
HCL	No	70 (47%)	0.512	49 (26.8%)	<0.001
	Yes	79 (53%)	0.512	134 (73.2%)	<0.001
HTG	No	77 (51.7%)	0.743	58 (31.7%)	<0.001
	Yes	72 (48.3%)	0.743	125 (68.3%)	<0.001
Dyslipidemia	No	93 (62.4%)	0.003	58 (31.7%)	<0.001
	Yes	56 (37.6%)	0.003	125 (68.3%)	<0.001
Obesity	No	113 (75.8%)	<0.001	66 (36.1%)	<0.001
	Yes	36 (24.2%)	<0.001	117 (63.9%)	<0.001
MetS	No	117 (78.5%)	<0.001	59 (32.2%)	<0.001
	Yes	32 (21.5%)	<0.001	124 (67.8%)	<0.001
Smoking	No	46 (30.9%)	<0.001	60 (32.8%)	<0.001
	Yes	103 (69.1%)	<0.001	123 (67.2%)	<0.001
ALC	No	116 (77.9%)	<0.001	122 (66.7%)	<0.001
	Yes	33 (22.1%)	<0.001	61 (33.3%)	<0.001
Coffee	No	12 (8.1%)	<0.001	16 (8.7%)	<0.001
	Yes	137 (91.9%)	<0.001	167 (91.3%)	<0.001
Drugs	No	149 (100%)	<0.001	183 (100%)	<0.001
Inflammation	No	88 (59.1%)	0.033	50 (27.3%)	<0.001
	Yes	61 (40.9%)	0.033	133 (72.7%)	<0.001
Tachycardia	No	87 (58.4%)	0.049	78 (42.6%)	0.054
	Yes	62 (41.6%)	0.049	105 (57.4%)	0.054
Genetic factors	No	31 (20.8%)	<0.001	67 (36.6%)	<0.001
	Yes	118 (79.2%)	<0.001	116 (63.4%)	<0.001
Absent risk factors	No	149 (100%)	<0.001	183 (100%)	<0.001

IHD—ischemic heart disease, HTN—hypertension, DM—diabetes mellitus, HCL—hypercholesterolemia, HTG—hypertriglyceridemia, MetS—metabolic syndrome, ALC—alcohol.

**Table 4:** Binomial test application for evaluating the impacts of symptoms on patients with depression alone versus depression with comorbid IHD.

		Depression = 149 Patients		Depression + IHD = 183 Patients	
Variable	Level	Counts	*p*	Counts	*p*
Low mood	No	12 (8.1%)	<0.001	4 (2.2%)	<0.001
	Yes	137(91.9%)	<0.001	179 (97.8%)	<0.001
Low energy	No	32 (21.5%)	<0.001	22 (12%)	<0.001
	Yes	117 (78.5%)	<0.001	161 (88%)	<0.001
Low activity	No	22 (14.8%)	<0.001	2 (1.1%)	<0.001
	Yes	127 (85.2%)	<0.001	181 (98.9%)	<0.001
Incapacity	No	59 (21.5%)	0.014	53 (29%)	<0.001
	Yes	90 (78.5%)	0.014	130 (71%)	<0.001
Uselessness	No	60 (40.4%)	0.021	42 (23%)	<0.001
	Yes	89 (59.7%)	0.021	141 (77%)	<0.001
Guilt	No	75 (50.3%)	1.000	93 (50.8%)	0.882
	Yes	74 (49.7%)	1.000	90 (49.2%)	0.882
Worthlessness	No	55 (36.9%)	0.002	49 (26.8%)	<0.001
	Yes	94 (63.1%)	0.002	134 (73.2%)	<0.001
Anhedonia	No	70 (47%)	0.512	47 (25.7%)	<0.001
	Yes	79 (53%)	0.512	136 (74.3%)	<0.001
Isolation	No	63 (42.3%)	0.071	9 (5%)	<0.001
	Yes	86 (57.7%)	0.071	174 (95%)	<0.001
Low self-esteem	No	55 (36.9%)	0.002	49 (26.8%)	<0.001
	Yes	94 (63.1%)	0.002	134 (73.2%)	<0.001
Rumination	No	31 (20.8%)	<0.001	25 (13.7%)	<0.001
	Yes	118 (79.2%)	<0.001	158 (86.3%)	<0.001
Lability	No	11 (7.4%)	<0.001	7 (3.8%)	<0.001
	Yes	138 (92.6%)	<0.001	176 (96.2%)	<0.001
Cognitive impairment	No	62 (41.6%)	0.049	43 (23.5%)	<0.001
	Yes	87 (58.3%)	0.049	140 (76.5%)	<0.001
Insomnia	No	50 (33.6%)	<0.001	51 (27.9%)	<0.001
	Yes	99 (66.4%)	<0.001	132 (72.1%)	<0.001
Low appetite	No	111 (74.5%)	<0.001	12 (6.6%)	<0.001
	Yes	38 (25.5%)	<0.001	171 (93.4%)	<0.001
Somatic symptoms	No	53 (35.6%)	<0.001	68 (37.2%)	<0.001
	Yes	96 (64.4%)	<0.001	115 (62.8%)	<0.001
Weight loss	No	111 (74.5%)	<0.001	12 (6.6%)	<0.001
	Yes	38 (25.5%)	<0.001	171 (93.4%)	<0.001
Low libido	No	67 (45%)	0.251	51 (27.9%)	<0.001
	Yes	82 (55%)	0.251	132 (72.1%)	<0.001
Suicidal thoughts	No	125 (83,9%)	<0.001	98 (53.6%)	0.375
	Yes	24 (16,1%)	<0.001	85 (46.4%)	0.375
Delusions	No	133 (89.3%)	<0.001	139 (76%)	<0.001
	Yes	16 (10.7%)	<0.001	44 (24%)	<0.001
Hallucinations	No	135 (90.6%)	<0.001	172 (94%)	<0.001
	Yes	14 (9.4%)	<0.001	11 (6%)	<0.001
Anxiety	No	62 (41.6%)	0.049	19 (10.4%)	<0.001
	Yes	87 (58.4%)	0.049	164 (89.6%)	<0.001

**Table 5:** Binomial test application for evaluating the impact of treatment, cardiological examination, and emergency admission hospitalization on patients with depression alone versus depression with comorbid IHD.

		Depression = 149 Patients		Depression + IHD = 183 Patients	
Variable	Level	Counts	*p*	Counts	*p*
Antidepressants	Yes	149 (100%)	<0.001	183 (100%)	<0.001
Antipsychotics	No	128 (85.9%)	<0.001	118 (64.5%)	<0.001
	Yes	21 (14.1%)	<0.001	65 (35.5%)	<0.001
Mood stabilizers	No	58 (38.9%)	0.008	41 (22.4%)	<0.001
	Yes	91 (61.1%)	0.008	142 (77.6%)	<0.001
Anxiolytics	No	62 (41.6%)	0.049	19 (10.4%)	<0.001
	Yes	87 (58.4%)	0.049	164 (89.6%)	<0.001
Hypnotic	No	50 (33.6%)	<0.001	51 (27.9%)	<0.001
	Yes	99 (66.4%)	<0.001	132 (72.1%)	<0.001
Other treatments	No	21 (14.1%)	<0.001	23 (12.6%)	<0.001
	Yes	128 (85.9%)	<0.001	160 (87.4%)	<0.001
Cardiological examination	No	75 (50.3%)	1.000	41 (22.4%)	<0.001
	Yes	74 (49.7%)	1.000	142 (77.6%)	<0.001
Emergency admissions	No	68 (45.6%)	0.326	75 (45.6%)	0.018
	Yes	81 (54.4%)	0.326	108 (54.4%)	0.018

## Data Availability

The raw data supporting the conclusions of this article will be made available by the authors on request.

## References

[1] GBD 2019 Diseases and Injuries Collaborators (2020). Global burden of 369 diseases and injuries in 204 countries and territories, 1990–2019: A systematic analysis for the Global Burden of Disease Study 2019. Lancet.

[2] Hooker S.A., O’Connor P.J., Sperl-Hillen J.M., Crain A.L., Ohnsorg K., Kane S., Rossom R. (2022). Depression and cardiovascular risk in primary care patients. J. Psychosom. Res..

[3] Amadio P., Zarà M., Sandrini L., Ieraci A., Barbieri S.S. (2020). Depression and Cardiovascular Disease: The Viewpoint of Platelets. Int. J. Mol. Sci..

[4] Cao H., Zhao H., Shen L. (2022). Depression increased risk of coronary heart disease: A meta-analysis of prospective cohort studies. Front. Cardiovasc. Med..

[5] Park D.H., Cho J.J., Yoon J.L., Kim M.Y., Ju Y.S. (2020). The Impact of Depression on Cardiovascular Disease: A Nationwide Population-Based Cohort Study in Korean Elderly. Korean J. Fam. Med..

[6] Lee C.H., Giuliani F. (2019). The Role of Inflammation in Depression and Fatigue. Front. Immunol..

[7] Severino P., D’Amato A., Pucci M., Infusino F., Adamo F., Birtolo L.I., Netti L., Montefusco G., Chimenti C., Lavalle C. (2020). Ischemic Heart Disease Pathophysiology Paradigms Overview: From Plaque Activation to Microvascular Dysfunction. Int. J. Mol. Sci..

[8] Gusev E., Sarapultsev A. (2023). Atherosclerosis and Inflammation: Insights from the Theory of General Pathological Processes. Int. J. Mol. Sci..

[9] Allabadi H., Probst-Hensch N., Alkaiyat A., Haj-Yahia S., Schindler C., Kwiatkowski M., Zemp E. (2019). Mediators of gender effects on depression among cardiovascular disease patients in Palestine. BMC Psychiatry.

[10] Monteleone P., Martiadis V., Maj M. (2011). Circadian rhythms and treatment implications in depression. Prog. Neuropsychopharmacol. Biol. Psychiatry.

[11] Crnko S., Du Pré B.C., Sluijter J.P.G., Van Laake L.W. (2019). Circadian rhythms and the molecular clock in cardiovascular biology and disease. Nat. Rev. Cardiol..

[12] Pivato C.A., Chandiramani R., Petrovic M., Nicolas J., Spirito A., Cao D., Mehran R. (2022). Depression and ischemic heart disease. Int. J. Cardiol..

[13] Sobolewska-Nowak J., Wachowska K., Nowak A., Orzechowska A., Szulc A., Płaza O., Gałecki P. (2023). Exploring the Heart–Mind Connection: Unraveling the Shared Pathways between Depression and Cardiovascular Diseases. Biomedicines.

[14] Bai B., Yin H., Guo L., Ma H., Wang H., Liu F., Liang Y., Liu A., Geng Q. (2021). Comorbidity of depression and anxiety leads to a poor prognosis following angina pectoris patients: A prospective study. BMC Psychiatry.

[15] Varghese T.P., Kumar A.V., Varghese N.M., Chand S. (2020). Depression Related Pathophysiologies Relevant in Heart Disease: Insights into the Mechanism Based on Pharmacological Treatments. Curr. Cardiol. Rev..

[16] Virzi N.E., Krantz D.S., Bittner V.A., Merz C.N.B., Reis S.E., Handberg E.M., Pepine C.J., Vaccarino V., Rutledge T. (2022). Depression Symptom Patterns as Predictors of Metabolic Syndrome and Cardiac Events in Symptomatic Women with Suspected Myocardial Ischemia: The Women’s Ischemia Syndrome Evaluation (WISE and WISE-CVD) Projects. Heart Mind Mumbai.

[17] Helmark C., Ahm R., Andersen C.M., Skovbakke S.J., Kok R., Wiil U.K., Schmidt T., Hjelmborg J., Frostholm L., Frydendal D.H. (2021). Internet-based treatment of anxiety and depression in patients with ischaemic heart disease attending cardiac rehabilitation: A feasibility study (eMindYourHeart). Eur. Heart J. Digit. Health.

[18] Borkowski P., Borkowska N. (2024). Understanding Mental Health Challenges in Cardiovascular Care. Cureus.

[19] Nielsen R.E., Banner J., Jensen S.E. (2021). Cardiovascular disease in patients with severe mental illness. Nat. Rev. Cardiol..

[20] Li X., Zhou J., Wang M., Yang C., Sun G. (2023). Cardiovascular disease and depression: A narrative review. Front. Cardiovasc. Med..

[21] Halma M., Plothe C., Marik P.E. (2024). Integrative Interventions for Improving Outcomes in Depression: A Narrative Review. Psychol. Int..

[22] Infurna F.J., Gerstorf D., Lachman M.E. (2020). Midlife in the 2020s: Opportunities and challenges. Am. Psychol..

[23] Aïdoud A., Gana W., Poitau F., Debacq C., Leroy V., Nkodo J.A., Poupin P., Angoulvant D., Fougère B. (2023). High Prevalence of Geriatric Conditions Among Older Adults with Cardiovascular Disease. J. Am. Heart Assoc..

[24] Zhao C., Lai L., Zhang L., Cai Z., Ren Z., Shi C., Luo W., Yan Y. (2021). The effects of acceptance and commitment therapy on the psychological and physical outcomes among cancer patients: A meta-analysis with trial sequential analysis. J. Psychosom. Res..

[25] Smith B., Netherway J., Jachyra P., Bone L., Baxter B., Blackshaw J., Foster C. (2022). Infographic. Communicate physical activity guidelines for disabled children and disabled young people. Br. J. Sports Med..

[26] Lee K.S., Hagan C.N., Hughes M., Cotter G., McAdam Freud E., Kircanski K., Leibenluft E., Brotman M.A., Tseng W.L. (2023). Systematic Review and Meta-analysis: Task-based fMRI Studies in Youths with Irritability. J. Am. Acad. Child Adolesc. Psychiatry.

[27] Korous K.M., Bradley R.H., Luthar S.S., Li L., Levy R., Cahill K.M., Rogers C.R. (2022). Socioeconomic status and depressive symptoms: An individual-participant data meta-analysis on range restriction and measurement in the United States. J. Affect. Disord..

[28] Izzi B., Tirozzi A., Cerletti C., Donati M.B., de Gaetano G., Hoylaerts M.F., Iacoviello L., Gialluisi A. (2020). Beyond Haemostasis and Thrombosis: Platelets in Depression and Its Co-Morbidities. Int. J. Mol. Sci..

[29] Jennings E.A., Chinogurei C., Adams L. (2022). Marital experiences and depressive symptoms among older adults in rural South Africa. SSM Ment. Health.

[30] Hsu M.-Y., Huang S.-C., Liu P.-L., Yeung K.-T., Wang Y.-M., Yang H.-J. (2022). The Interaction between Exercise and Marital Status on Depression: A Cross-Sectional Study of the Taiwan Biobank. Int. J. Environ. Res. Public Health.

[31] Herrera P.A., Campos-Romero S., Szabo W., Martínez P., Guajardo V., Rojas G. (2021). Understanding the Relationship between Depression and Chronic Diseases Such as Diabetes and Hypertension: A Grounded Theory Study. Int. J. Environ. Res. Public Health.

[32] Berk M., Köhler-Forsberg O., Turner M., Penninx B.W.J.H., Wrobel A., Firth J., Loughman A., Reavley N.J., McGrath J.J., Momen N.C. (2023). Comorbidity between major depressive disorder and physical diseases: A comprehensive review of epidemiology, mechanisms and management. World Psychiatry.

[33] Carlson D.M., Yarns B.C. (2023). Managing medical and psychiatric multimorbidity in older patients. Ther. Adv. Psychopharmacol..

[34] Rawashdeh S.I., Ibdah R., Kheirallah K.A., Al-Kasasbeh A., Raffee L.A., Alrabadi N., Albustami I.S., Haddad R., Ibdah R.M., Al-Mistarehi A.H. (2021). Prevalence Estimates, Severity, and Risk Factors of Depressive Symptoms among Coronary Artery Disease Patients after Ten Days of Percutaneous Coronary Intervention. Clin. Pract. Epidemiol. Ment. Health.

[35] Brown L.C., Stanton J.D., Bharthi K., Maruf A.A., Müller D.J., Bousman C.A. (2022). Pharmacogenomic Testing and Depressive Symptom Remission: A Systematic Review and Meta-Analysis of Prospective, Controlled Clinical Trials. Clin. Pharmacol. Ther..

[36] Buysse D.J., Angst J., Gamma A., Ajdacic V., Eich D., Rössler W. (2008). Prevalence, course, and comorbidity of insomnia and depression in young adults. Sleep.

[37] Moreno V., Dévora S., Abdala-Kuri S., Oliva A. (2023). Trends in the Consumption of Antidepressant Drugs before and during the COVID-19 Pandemic in the Canary Islands, Spain: The Case of the Province of Las Palmas. Healthcare.

[38] Dobrek L., Głowacka K. (2023). Depression and Its Phytopharmacotherapy—A Narrative Review. Int. J. Mol. Sci..

[39] Rutledge T., Redwine L.S., Linke S.E., Mills P.J. (2013). A meta-analysis of mental health treatments and cardiac rehabilitation for improving clinical outcomes and depression among patients with coronary heart disease. Psychosom. Med..

[40] Fang S., Zhang W. (2024). Heart–Brain Axis: A Narrative Review of the Interaction between Depression and Arrhythmia. Biomedicines.

[41] Aljunaid M.A., Alosaimi R.M., Alazmi E.A., Afandi A.A., Musslem M.T., Aljarameez M.M., Alzobaidi H.H. (2024). Determinants of Depression in Caregivers of Geriatric Patients in Jeddah, Saudi Arabia: A Cross-Sectional Study. Medicina.

